# Incidence of surgical procedures for gastrointestinal complications after abdominal wall closure in patients with gastroschisis and omphalocele

**DOI:** 10.1007/s00383-021-04977-0

**Published:** 2021-08-25

**Authors:** M. Haghshenas, U. Rolle, M. Hutter, T. M. Theilen

**Affiliations:** Department of Pediatric Surgery and Pediatric Urology, Goethe-University Frankfurt, University Hospital Frankfurt, Theodor-Stern-Kai 7, 60590 Frankfurt, Germany

**Keywords:** Ileus, Bowel obstruction, Parenteral nutrition, Short bowel syndrome, Trisomy 21, Beckwith–Wiedemann syndrome

## Abstract

**Purpose:**

This study aims to define the extent of additional surgical procedures after abdominal wall closure (AWC) in patients with gastroschisis (GS) and omphalocele (OC) with special focus on gastrointestinal related operations.

**Methods:**

A retrospective chart review was performed including all operations in GS and OC patients in the first year after AWC (2010–2019). The risk for surgery was calculated using the one-year cumulative incidence (CI).

**Results:**

33 GS patients (18 simple GS, 15 complex) and 24 OC patients (12 without (= OCL), 12 OC patients with liver protrusion (= OCL +)) were eligible for analysis. 43 secondary operations (23 in GS, 20 in OC patients) occurred after a median time of 84 days (16–824) in GS and 114.5 days (12–4368) in OC. Patients with complex versus simple GS had a significantly higher risk of undergoing a secondary operation (one-year CI 64.3% vs. 24.4%; *p* = 0.05). 86.5% of surgical procedures in complex GS and 36.3% in OCL + were related to gastrointestinal complications. Complex GS had a significantly higher risk for GI-related surgery than simple GS. Bowel obstruction was a risk factor for surgery in complex GS (one-year CI 35.7%).

**Conclusion:**

Complex GS and OCL + patients had the highest risk of undergoing secondary operations, especially those with gastrointestinal complications.

**Supplementary Information:**

The online version contains supplementary material available at 10.1007/s00383-021-04977-0.

## Introduction

The two most common congenital wall defects are gastroschisis (GS) and omphalocele (OC). Depending on the complexity of the abdominal wall defect and concomitant malformations surgical abdominal wall closure is a single or multiple step approach [1, 2]. In cases of multistage closure and surgical repairs of associated malformations, patients are at risk of accumulating a higher number of operative procedures in their early days of life. This is especially relevant, as such operations carry the risk for repeated and prolonged anesthesia as well as prolonged intensive care stay. After abdominal wall closure, the load of operations can even increase in cases of secondary complications with the need for surgical interventions [3].

Concomitant complications in GS are frequent. Up to 30% of patients present initially with a complex GS revealing bowel atresia or bowel stenosis [4, 5]. A subset of patients also suffers from secondary diseases such as transient or sustained bowel dysmotility, necrotizing enterocolitis (NEC; 3.8–8.2%), volvulus (0.5–3.0%), intestinal necrosis (4.5%), and vanishing gastroschisis (< 1.0%) [5–9]. These conditions can lead to long-term complications such as intolerance of enteral feeds, short gut syndrome (3.2–11.4%), intestinal failure, and adhesive bowel obstruction (20.4–27.0%) [3–5, 10, 11].

Complications in patients with OC are associated with structural organ defects, special anatomical conditions, and genetic alterations. In the case of liver protrusion into the omphalocele (OCL +), hepatic congestion can occur due to liver vein kinking after repositioning the liver into the abdominal cavity. Abdominal wall closure can lead to an abdominal compartment syndrome and kidney failure. The main predominate for morbidity in OC are congenital structural organ defects such as severe cardiac defects (18.0–32.0%), pulmonary hypoplasia (9.6%), diaphragmatic defects (15%), or medical conditions such as pulmonary hypertension (8.3–57.0%) [11–15]. Chromosomal and gene abnormalities are found in 20–50% of patients and are mostly associated with omphaloceles without liver protrusion (OCL-). Beckwith–Wiedemann syndrome and trisomies 13, 18, and 21 are the most prevalent associated genetic alterations, which also carry the risk of reduced survival [16–18]. Overall, isolated OC without any structural or genetic abnormalities only occurs at a rate of 10–14% [19].

All of the above-mentioned modalities carry the risk for further surgical procedures contributing to the prognosis of patients with abdominal wall defects [20, 21]. However, data on the incidences and outcomes of secondary operative procedures after abdominal wall closure are sparse. Therefore, the aim of this study was to retrospectively investigate the rate, indication, timing, and outcome of surgical procedures after abdominal wall closure. The main focus was on gastrointestinal complications requiring surgical treatment.

## Methods

### Patients

We conducted a retrospective review of all liveborn patients diagnosed with GS and OC between 2010 and 2019 at our institution. A total of 61 patients (35 patients with GS and 26 patients with OC) were identified.

At our institution, due to the established protocol, all patients with GS and OC were delivered by scheduled cesarean section. Premature delivery was due to maternal or fetal complications such as HELLP syndrome, preeclampsia, premature labor, pathological cardiotocography, and bowel wall thickening of the protruded bowel in prenatal ultrasound examinations. All patients were born in our hospital except for four patients with OCL- and one patient who was referred to us on the day of delivery with a complex GS, including protrusion of the liver and spleen. The latter received a Schuster’s abdominoplasty but died on day four of life due to cardiorespiratory failure. This patient and another patient with simple GS who were lost to follow-up after transfer to another hospital were omitted from the analysis of the surgical procedures after abdominal wall closure. Among patients with OC, one patient who had OCL- with arthrogryposis multiplex congenita and diaphragmatic hernia was excluded from further analysis due to lethal complications on day one of life (hydrops fetalis with respiratory failure). Another patient with OCL-, trisomy 13, and tetralogy of Fallot was treated palliatively after birth and was also excluded from further analysis.

### Surgical management

Cesarean section was the primary delivery mode according to the protocol of our perinatal center including obstetrics, neonatology, and pediatric surgery. After postnatal adaptation patients were taken to the operation theater within 3–4 h after delivery. All patients received routinely a peripheral central venous access (PICC line). In all cases in which abdominal wall closure was not achievable due to increased abdominal pressure leading to respiratory or cardiocirculatory compromise patients underwent staged abdominal wall closure (patch or silo abdominoplasty). Open heart operations for congenital heart defects were performed at an affiliated pediatric cardiac surgery center whereas heart catheter interventions were performed in-house. All interventions were performed under general anesthesia.

### Outcome measures

Complex GS was defined as GS with bowel alterations causing bowel stenosis, bowel atresia, bowel perforation, or volvulus (bowel ischemia), as defined by Molik et al. [20].

There is no clear consensus regarding the classification of OCs. Multiple criteria have been applied, such as inability to achieve primary closure, diameter of the sac or the abdominal wall, contents of the sac, and volume disproportion [22, 23]. We divided all OCs into OSs without (OCL −) or with protrusion of the liver (OCL +) into the sac [22]. OCL − cases resembled minor OCs and OCL + cases resembled giant OCs in our cohort. We found this classification useful as the defects could be subdivided clinically at the bedside.

Data from medical and surgical records were extracted for sex, gestational age, birth weight, type of abdominal wall defect, associated structural and genetic defects, primary surgical treatment, additional surgical procedures after abdominal wall closure, time to full enteral feeding, length of hospital stay, mortality, gastrointestinal complications, and follow-up time.

Gastrointestinal complications were counted whenever surgery was needed for intestinal reasons (Hickman catheter placement for parenteral nutrition, gastrostomy placement for continuous enteral nutrition), for bowel revisions (such as adhesive ileus), for the treatment of secondary intestinal diseases (gastroesophageal reflux), and for intestinal diagnostics (such as rectal biopsy for Hirschsprung’s disease).

### Statistical analysis

Continuous variables are displayed as the means and standard deviations (SDs), and nonnormally distributed data are expressed as medians and ranges. Statistical significance was calculated by the Mann–Whitney *U* test and Fisher’s exact test for means and proportions, respectively. The one-year cumulative incidence (CI) was calculated to estimate the risk of undergoing additional surgery after abdominal wall closure. A *p* value less than 0.05 was considered significant. For estimating the mean number operations per patient the one-year mean cumulative count was calculated [24]. All data were processed for statistical analysis using R software (version 3.4.0).

### Ethical approval

The study was approved by the Institutional Ethics Committee of the University Hospital Frankfurt, Goethe University Frankfurt (Approval No. 277-18).

## Results

### Patient characteristics

We identified 35 patients with GS. Nineteen of 35 patients (54.3%) had a simple GS and 16/35 patients (45.7%) had a complex GS (Table 1). Five patients had additional congenital structural heart defects (one patient with ventricular septum defect, one patient with tricuspid insufficiency, three patients with atrial septum defect), two patients had skin appendages, one patient had unilateral hydronephrosis, and another patient had scaphocephaly.

**Table 1:** Characteristics and postnatal clinical course of 35 patients with gastroschisis and 26 patients with omphalocele

	Gastroschisis			Omphalocele		
	Simple	Complex		w/o liver protrusion	With liver protrusion	
Number of patients	19	16	–	14	12	–
Sex (m/f)	11/8	6/10	–	7/5	7/5	–
Median age at birth in days (range)	249 (235–271)	245 (229–268)	0.116	263 (246–284)	260 (196–275)	0.875
Average body weight at birth in grams (± SD)	2375 (401)	2241 (556)	0.103	2903 (737)	2512 (549)	0.129
Preterm delivery < 37th week of gestation (%)	12 (63)	14 (88)	0.135	4 (29)	6 (50)	0.421
Abdominal wall closure						
Primary (%)	12 (63.2)	5 (31.3)	–	12 (85.7)	4 (33.3)	–
Staged (%)	7 (36.8)	10 (62.5)		–	8 (66.7)	
No closure (%)	–	1 (6.3)^a^		2 (14.3)^b^	–	
Median number of operations for abdominal wall closure (range)	1.0 (1.0–3.0)	1.5 (1.0–5.0)	–	1.0	1.0 (1.0–4.0)	–
Median time of parenteral nutrition in days (range)	17.5 (7.0–97.0)	22.0 (3.0–201.0)	0.940	7.0 (3.0–35.0)	32.0 (2.0–89.0)	0.037
Median time to full feeds in days (range)	26.00 (2.0–137.0)	24.00 (5.0–258.0)	0.602	11.00 (5.0–151.0)	24.50 (15.0–196.0)	0.007
Median time of hospital stay in days (range)	31.5 (2.0–137.0)	28.0 (5.0–258.0)	0.304	13.5 (5.0–151.0)	38.0 (15.0–196.0)	0.001
Median follow-up in days (range)	491 (20.0–2,754.0)	199 (6.0–2,930.0)	0.974	124 (4.0–4368.0)	868 (105.0–3082.0)	0.063

*w/o* without, *SD* standard deviation

^a^One patient died with a silicone silo in situ

^b^Two patients died before abdominal wall closure

There were 26 patients with OC of whom 14 patients had OCL− and 12 patients had OCL + . Seven of 26 patients (26.9%) suffered from OC in association with genetic abnormalities, such as trisomy 18 (one patient), trisomy 21 (two patients), Beckwith–Wiedemann syndrome (three patients), and Arthrogryposis multiplex congenita (one patient). In addition, multiple associated structural defects were also seen among patients with OC. The most frequent were heart defects in 14/26 patients (53.8%; seven patients with atrial septum defects, three with ventricular septum defects, two with tetralogy of Fallot, one with stenosis of the pulmonary artery, and one with bicuspid aortic valve). Other clinically relevant associated malformations were diaphragmatic hernia (3/26 patients (11.5%)), pulmonary hypoplasia (2/26 patients (7.7%)), pulmonary hypertension (2/26 patients (7.7%)), unilateral renal dysplasia, primary obstructive megaureter, microtia III°, microcephaly, and pes equinovarus (each 1/26 patients (3.8% each)).

Table 1 summarizes the disease associated characteristics of all GS and OC patients. While there were no significant differences between simple and complex GS in terms of duration of parenteral nutrition and time to full feeds, these two variables were significantly different between patients with OCL − and OCL + (*p* = 0.037 and *p* = 0.007, respectively). In addition, patients with OCL + had a significantly longer length of hospital stay than patients with OCL − (*p* = 0.001; Table 1).

### Abdominal findings at primary surgery

We identified 15/35 GS patients (42.9%) with bowel malformations during the primary surgical repair. Bowel atresia was the most prominent abdominal finding, affecting 9/35 patients (25.7%). In 6/35 patients (17.1%), mesenteric bands and bands of a remnant omphalomesenteric duct (Meckel’s band) were identified. Four of these bands caused atresia or bowel stenosis (Table 2). Bowel perforation occurred in one patient due to preatretic dilatation of the small bowel. Another patient suffered from severe traumatic birth injury with semicircumferential tears of the esophageal-gastric junction, stomach, and jejunum due to traumatic cesarian section. Two patients showed bowel findings suspicious for cystic fibrosis (ileal meconium congestion with unused colon distally) and Hirschsprung’s disease (megacolon). The patient with megacolon received a temporary enterostomy. In both cases, later diagnostics did not confirm the suspected diagnoses (Supplementary Table 1).

**Table 2:** Surgical procedures during the first year after abdominal wall closure in 33 patients with gastroschisis and 24 patients with omphalocele

Number of operative procedures (%)	Gastroschisis		Omphalocele	
	Simple *n* = 5	Complex *n* = 22	w/o liver protrusion *n* = 3	With liver protrusion *n* = 22
Gastrointestinal-related procedures	2 (40.0)	19 (86.5)	1 (33.3)	8 (36.3)
Relaparotomy for				
Adhesive bowel obstruction	2 (40.0)	6 (27.4)	–	
Small bowel volvulus	–	1 (4.5)	–	–
Abdominal infection	–	1 (4.5)	–	–
Mesentery bleeding	–	1 (4.5)	–	–
Enterostomy closure	–	3 (13.7)	–	–
Anastomotic leak	–	–	–	1 (4.5)
Iatrogenic rectal perforation	–	–	–	1 (4.5)
Enterostomy formation	–	–	–	1 (4.5)
Hickman catheter^a^				
Implantation	–	4 (18.3)	–	2 (9.1)
Removal	–	2 (9.1)	–	–
Gastrostomy	–	–	–	1 (4.5)
Gastric fundoplication^b^	–	–	1 (33.3)	2 (9.1)
Rectal biopsy to rule out HD	–	1 (4.5)	–	–
Airway-related procedures	–	–	–	1 (4.5)
Tracheostomy^c^				1 (4.5)
Cardiac procedures	–	–	–	5 (22.9)
Interventional cardiac catheterization				3 (13.9)
Open VSD/ASD patch closure				1 (4.5)
Tetralogy of Fallot repair				1 (4.5)
Other procedures	3 (60.0)	3 (13.5)	2 (66.6)	8 (36.3)
Umbilical hernia repair	1 (20.0)	–	–	–
Inguinal hernia repair	2 (40.0)	2 (9.0)	–	5 (22.9)
Hydrocele resection of the testis	–	–	–	1 (4.5)
Orchidopexy	–	–	1 (33.3)	1 (4.5)
Surgical repair of scaphocephaly	–	1 (4.5)	-	–
Ureterocystoneostomy	–	–	1 (33.3)	–
Resection of ear tag	–	–	–	1 (4.5)

*w/o* without, *HD* Hirschspung’s disease, *VSD* ventricular septum defect

^a^For prolonged parenteral nutrition

^b^For gastroesophageal reflux disease

^c^Lung hypoplasia of a patient with initial diaphragmatic hernia

There were 8/26 OC patients (30.8%) with additional abdominal abnormalities diagnosed during the primary repair. Six patients (23.1%) had a patent omphalomesenteric duct and two patients (7.7%) had a urachal fistula inserting into the umbilical cord structures. Three of 26 patients (11.5%) had a diaphragmatic hernia (Supplementary Table 1).

### *Secondary surgical procedures* < *one year after abdominal wall closure*

23/25 operations (92.0%) in GS patients and 20/24 operations (83.3%) in OC patients performed within the first year after abdominal wall closure. To standardize the results, we only considered surgical procedures up to one year after abdominal wall closure in our analysis.

**Fig. 1:**
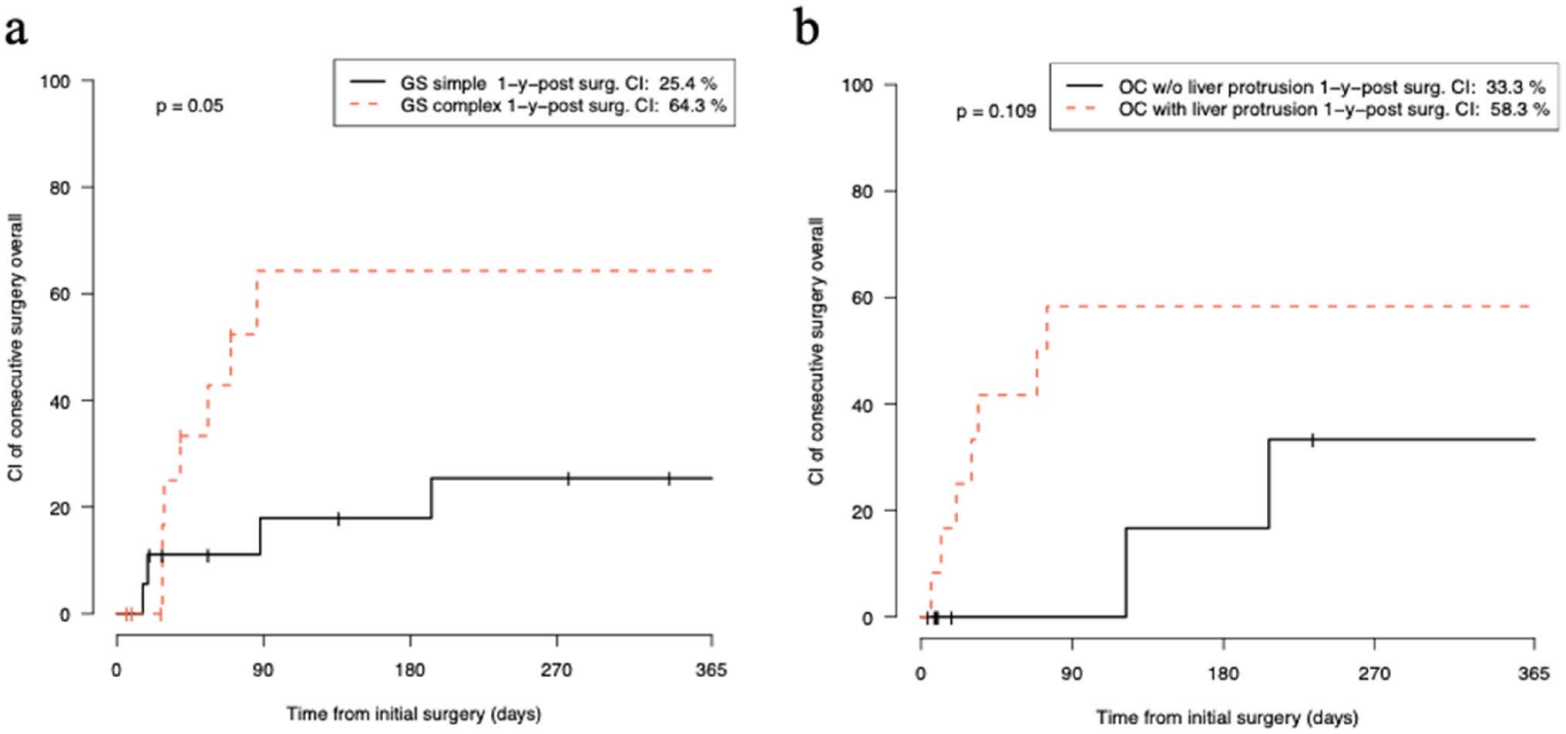
Kaplan–Meier curve showing the one-year cumulative incidence of consecutive surgeries after initial surgery for abdominal wall closure in patients with gastroschisis **a** and omphalocele **b**. 1-y-post surg. *CI* one-year cumulative incidence after initial surgery for abdominal wall closure

We identified 11/33 GS patients (4/18 patients with simple GS and 7/15 patients with complex GS) who received one or more operations during the first year after abdominal wall closure. Patients with a complex type of GS had a significantly higher risk for an additional surgical procedure than patients with a simple type of GS (one-year CI 64.3% vs. 25.4%, *p* = 0.05; Fig. 1a).

Among patients with OC, the number of patients receiving surgical procedure (9/24 patients) after abdominal wall closure was not significantly different between patients with (7/12 patients) and without liver protrusion (2/12 patients; one-year CI 58.3% vs. 33.3%, *p* = 0.109) during the first year after abdominal wall closure (Fig. 1b).

**Fig. 2:**
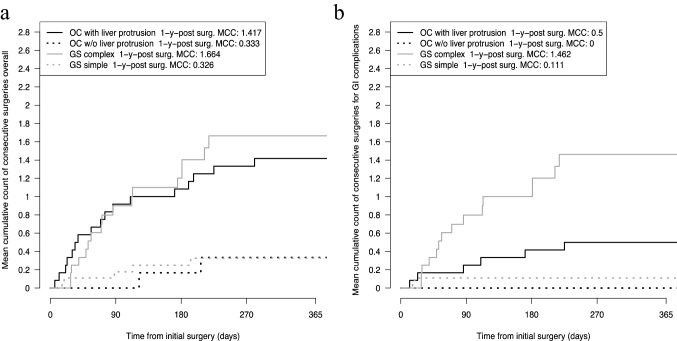
Estimated mean number of surgeries overall **a** and of surgeries for gastrointestinal complications **b** per patient with gastroschisis and omphalocele during the first year after initial surgery for abdominal wall closure. *GS* gastroschisis, *OC* omphalocele, *GI* gastrointestinal, 1-y-post surg. *MCC* one-year mean cumulative count after initial surgery for abdominal wall closure, *w/o* without

Overall, 11 patients with GS underwent 23 secondary surgical procedures (five in simple GS and 18 in complex GS), leading to mean cumulative counts (MCC) of 0.326 operations per patient and year in simple GS and 1.664 in complex GS (Fig. 2a). These operations included 27 different procedures (five in simple GS and 22 in complex GS; Table 2).

In the cohort of patients with OC, 20 operations (three in OCL − and 17 in OCL +) were performed during the first year after abdominal wall closure. In these operations, 25 procedures were performed (three in OCL − and 22 in OCL +). This led to MCCs of 0.333 and 1.417 operations per patient and year, respectively (Fig. 2a).

### Secondary surgical procedures for gastrointestinal complications

The majority of surgical procedures were related to gastrointestinal conditions, i.e., relaparotomy for different bowel procedures, Hickman catheter insertion for parenteral nutrition in short bowel syndrome, gastrostomy placement for enteral feeding support, gastric fundoplication for gastroesophageal reflux, and rectal biopsy to rule out Hirschprung’s disease. Table 2 lists all procedures performed in GS and OC patients during the first year after abdominal wall closure.

**Fig. 3:**
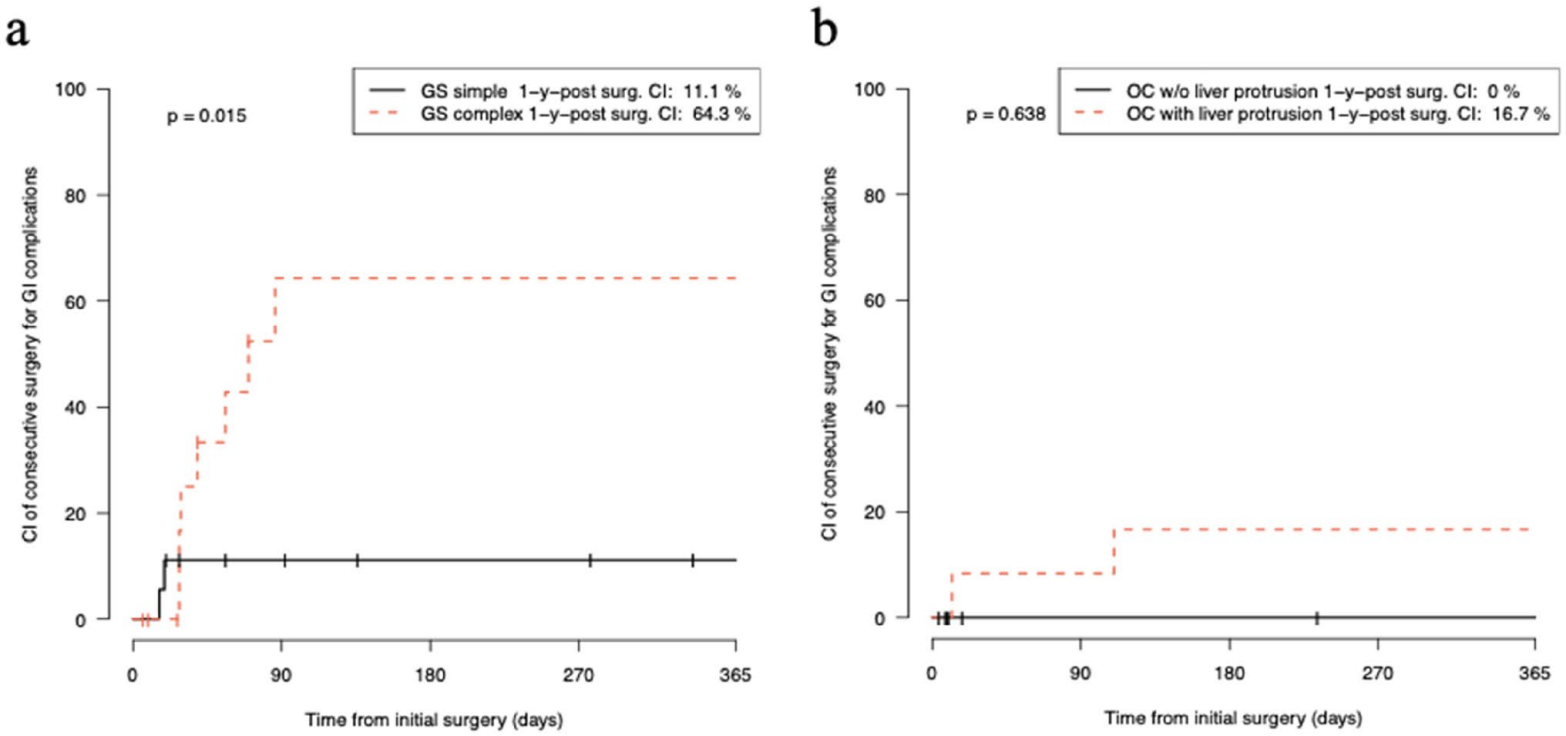
One-year cumulative incidence of consecutive operations for gastrointestinal complications after initial surgery for abdominal wall closure in patients with gastroschisis **a** and omphalocele **b**. 1-y-post surg. *CI* one-year cumulative incidence after initial surgery for abdominal wall closure, *GS* gastroschisis, *OC* omphalocele, *w/o* without

Patients with complex GS had a significantly higher risk of undergoing secondary surgery for gastrointestinal complications within the first year after abdominal wall closure than patients with simple GS (one-year CI 64.3% vs. 11.1%, *p* = 0.015, Fig. 3a).

Comparison of the two types of OC revealed that there was no difference in the one-year CI for surgical procedures related to gastrointestinal complications (one-year CI 0% vs. 16.7%, *p* = 0.638; Fig. 3b).

In GS, 17/23 operations (74.0%) were performed in relation to gastrointestinal complications (two in simple GS and 15 in complex GS), leading to MCCs of 0.111 operations in simple GS and 1.462 in complex GS per patient and year (Fig. 2b). These operations included 19 different surgical procedures in complex GS and 2 in simple GS (Table 3).

**Table 3:** List of patients with gastroschisis and omphalocele who died before and after abdominal wall closure

Abdominal wall defect	Age at death in days	Successful AWC	Cause of death	Prematurity	Previous history/preexisting conditions
Complex GS	4	No	Respiratory failure with Schuster’s abdominoplasty	Yes GA = 32 weeks BW1250g	GS with protruding liver and spleen, admission on first day of life after birth in another hospital
Complex GS	64	Yes	Organ failure in the course of acute small bowel volvulus, mesentery bleeding and mass blood transfusion	Yes GA = 33 weeks BW = 2200 g	Apple peel atresia, undefined congenital hepatopathy with bleeding disorder
Complex GS	251	Yes	TPN- associated liver failure	Yes GA = 33 weeks BW = 2170 g	Small bowel perforation with long-segment bowel atresia, one reoperation for adhesive small bowel obstruction, short bowel syndrome
OCL −	0	No	Respiratory failure, hydrops fetalis		Arthrogryposis multiplex congenita
OCL +	106	Yes	Respiratory and cardiac failure, pulmonary hypertension with right ventricular overload	Yes GA = 28 weeks, BW = 1480 g	Meconium ileus, Beckwith–Wiedemann Syndrome, broncho-pulmonary dysplasia, thrombophilia with thrombus of V. cava
OCL −	209	No^a^	Acute pneumonia	No GA = 37 weeks BW = 2190 g	Trisomie 13, Tetralogy of Fallot
OCL +	294	Yes	Respiratory failure	No GA 37 weeks BW = 1815 g	Diaphragmatic hernia, pulmonary hypoplasia, ciliary dysfunction of the lung, recurrent pneumonia, gastric fundoplication, tracheostomy
OCL +	422	Yes	Influenza A pneumonia, died with veno-venous ECMO	Yes GA = 33 weeks BW = 2230 g	Fallot repair, dysphagia, Gastrostomy placement

*AWS* abdominal wall closure, *GS* gastroschisis, *GA* gestational age, *BW* birth weight, *OC* omphalocele, *TPN* total parenteral nutrition, *ECMO* extracorporeal membrane oxygenation

^a^This patient did not receive closure of the omphalocele after birth and was treated palliatively, the patient died due to pneumonia at the age of 209 days

In OC, 6/20 operations (30.0%) were needed for gastrointestinal complications (zero in OCL − (one-year MCC: 0) and six in OCL + (one-year MCC: 0.500); Fig. 2b). There were eight procedures in OCL + patients and one in OCL-patients (Table 2).

Considering only reoperations for adhesive bowel obstruction, patients with complex GS were more frequently affected than patients with simple GS. However, this trend did not reach a significant difference (one-year CI 35.7% vs. 11.1%, *p* = 0.222).

There was only one operation performed for bowel obstruction among the OC patients. This operation occurred 8.1 years after AWC. During the first year after AWC, no operation was performed for bowel obstruction in OC patients.

### Time interval of operative procedures after abdominal wall closure

The median times to a reoperation after abdominal wall closure were 84 days (16–824 days) in GS patients and 114.5 days (12–4368 days) in OC patients.

### *Other surgical procedures* < *one year after abdominal wall closure*

Patients who previously had an OCL + had a high incidence of inguinal hernia repair (5/22 procedures (22.9%)) and cardiac procedures (5/22 procedures (22.9%)). Of note, another three procedures for inguinal hernia repair in OCL + patients were performed later than 1 year after abdominal wall closure, as stated above. Table 2 lists all the operative procedures.

### *Secondary surgical procedures* > *1 year after abdominal wall closure*

There were only two surgical procedures in patients with GS (one umbilical hernia repair in simple GS and one orchidopexy in complex GS) that occurred later than one year after abdominal wall closure. Among OC patients, four surgical procedures were performed later than one year after abdominal wall closure (one testicular biopsy in case of testicular mass in OCL-, three inguinal hernia repairs and one operation for bowel obstruction in OCL +).

### Mortality after abdominal wall closure

**Fig. 4:**
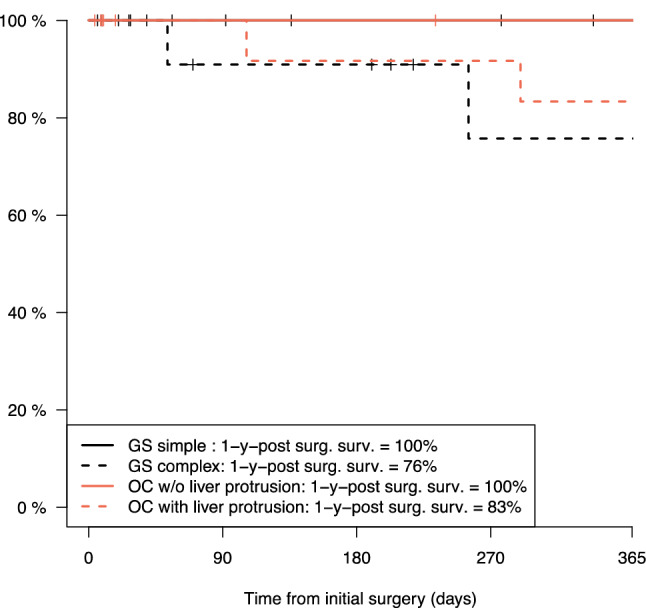
Mortality after initial surgery for abdominal wall closure in gastroschisis and omphalocele. 1-y-post surg. *surv.* one-year survival after initial surgery for abdominal wall closure, *GS* gastroschisis, *OC* omphalocele, *w/o* without

Within the first year after abdominal wall closure, two patients died who had complex GS (2/15 patients, 76% one-year survival for patients with complex GS) and two patients died who had OCL + (2/12 patient, 83% one-year survival for patients with OCL + ; Fig. 4). The survival of patients was not statistically different between simple GS and complex GS (*p* = 0.059) as well as between OCL − and OCL + patients (*p* = 0.228).

After abdominal wall closure, only patients with complex GS died due to gastrointestinal-related complications. Mortality among patients with OC was only due to respiratory- and cardiac-related diseases. Table 3 lists all mortality cases that occurred before and after abdominal wall closure.

The overall mortality rates, including deaths before and after abdominal wall closure as well as deaths of patients who were initially excluded from analysis because they did not receive surgery for abdominal wall closure, were zero in simple GS, 3/16 patients (18.7%) in complex GS, 2/14 patients (14.9%) with OCL −, and 3/12 patients (25.0%) in OCL + .

## Discussion

In this study, we reviewed the incidence of secondary surgical procedures after abdominal wall closure in patients with GS and OC in our institution. Overall, the proportion of patients with secondary operations in our cohort was almost equal between GS and OC (33.3% vs. 37.5%). In the literature, there are only a few data on secondary operations in abdominal wall defects. In GS, the rate for secondary operations has been reported to range from 11.7 to 25.9% [3, 25]. In these publications, however, only secondary operations directly related to bowel or abdominal wall complications were analyzed. When we extracted these indications for surgery from our data, we found rates of 21.2% for GS patients (11.1% in simple GS and 33.3% in complex GS) and 6.2% for OC patients (12.5% in OCL + , none in OCL − patients) in the first year after abdominal wall closure.

Most surgical procedures in patients with GS and OC are reported to occur during the first year after abdominal wall closure. Among our patients, more than 92% of operations in GS and 83% in OC were performed within the first year. Other authors have also documented early surgical intervention in the first year following gastroschisis closure for bowel and abdominal wall complications (92%) and cases of adhesion ileus (87%) [3, 26].

In our study, we estimated the risk of undergoing surgery after abdominal wall closure by calculating the one-year cumulative incidence. A total of 64.3% of patients with complex GS carried a risk of having at least one secondary operation within the first year after gastroschisis closure. This rate was significantly higher than that of patients with simple GS (24.4%). Among OC patients, OCL − and OCL + did not show a significant difference in the risk of undergoing surgery (58.3% vs. 33.3%, respectively) in the first year after abdominal wall closure.

A greater burden for surgical interventions in patients with complex GS has also been shown by others. Friedmacher and colleagues calculated a five times higher relative risk of undergoing a secondary operation for indications related to bowel or abdominal wall complications in patients with complex GS compared to simple GS [3]. By definition, patients with complex GS suffer from bowel pathologies such as atresia, stenosis, and necrotizing or vanishing bowel, which may cause long-term gastrointestinal complications and eventually leading to secondary surgical interventions.

In our cohort, 74.0% of surgical procedures in GS and 30.0% in OC were related to gastrointestinal complications. These procedures consisted of acute emergency operations such as bowel obstructions, bowel perforation, or mesentery bleeding as well as elective procedures such as enterostomy closure, implantation of Hickman catheters, gastrostomy placement for enteral nutrition in short bowel syndrome, gastric fundoplication for gastroesophageal reflux, and rectal biopsy for suspected Hirschsprung’s disease.

### Bowel obstruction

One of the acute gastrointestinal complications is an adhesive bowel obstruction with the need for abdominal revision surgery. We found a one-year CI of 21.5% in GS (35.7% in complex vs. 11.1% in simple GS). All patients needed surgery within the first year of life. In OC, there was only one patient with a laparotomy for ileus occurring 8.1 years after abdominal wall closure. In the literature, the incidence rates of small bowel adhesion are reported to be between 6.25 and 27.0% in GS and approximately 13% in the first year of life in OC [3, 10, 26–28]. However, the incidence of a bowel obstruction leading to an operation extends beyond the first year of life. Van Eijck and colleagues reported an incidence of 37% of small bowel obstruction related to GS in the first 10 years [10]. Others have reported an incident of small bowel obstruction after GS repair in 30% of cases after the second year of life and even in up to 30% of cases after the age of 16 [29, 30]. The follow-up time in our study was too short to confirm these findings in our patients.

Another reason for bowel obstruction is a small bowel volvulus. Patients with GS have non- or malrotated bowel fixation, the latter carrying a risk for small bowel volvulus. We had one GS patient (3.0%) with a 180° volvulus who died due to a bleeding disorder and organ failure. In the literature, the incidence of small bowel volvulus is reported to be between 0.5 and 3.0% [8, 9]. Parents of children with GS and OC need to be counseled for signs and symptoms of a volvulus with the need for emergent revisits.

### Short bowel syndrome

Complex GC is highly associated with short bowel syndrome [5, 6]. In our cohort, 2/16 patients with complex GS had short bowel syndrome due to complex bowel atresias. They needed surgical placement for Hickman catheters for long-term parenteral nutrition. One patient died due to liver intoxication, and the other patients could eventually be weaned from parenteral nutrition. Reasons for loss of bowel in GS are complex atresias, recurrent surgery for bowel obstruction, necrotizing enterocolitis, or vanishing gastroschisis. Although patients with complex GS are reported to have an increased risk for NEC, we did not observe a single case in our cohort [6, 7].

Other indications for Hickman catheters for temporary parenteral nutrition and medication in addition to short bowel syndrome in our cohort were long-term intensive care treatment with recurrent operations in two patients with complex GS and in two patients with OCL + .

### Gastroesophageal reflux

Gastroesophageal reflux (GER) is common in patients with abdominal wall defects, occurring in up to 70% [7, 31, 32]. Among multiple other factors, hiatal hernia contributes to the pathology of GER disease (GERD). Approximately, 11–19% of patients with GS are reported to have a hiatal hernia in association with GERD [5, 33]. In our series, however, none of the GS patients had clinical evidence of a hiatal hernia with pathologic GERD and the need for anti-reflux surgery.

Among OC patients, relevant GER has been reported especially in patients with large OC defects [31, 34]. In our cohort of OC patients, one patient with a small defect (OCL −) and two with larger defects (OCL +) had severe GERD without radiographic signs of hiatal hernia. Gastric fundoplication restored feeding capability in these patients.

Overall, however, GER in patients with abdominal wall defects is reported to respond well to medical treatment and shows a clear tendency toward spontaneous improvement within the first years of life [5, 32, 34]. Anti-reflux surgery should therefore only be considered for severe cases of GER, GER on the basis of a hiatal hernia, and failure of medical treatment.

### Mortality

We observed a substantial rate of mortality after abdominal wall closure in complex GS and OCL + patients. Two patients with complex GS died of acute gastrointestinal complications. Although survival has improved significantly over the last decades in GS patients, late mortality is frequently reported. A single-center study from Denmark reported relevant late mortality (after the neonatal period) related to parenteral nutrition associated liver failure (complex GS) and small bowel obstruction (simple GS) [35]. The reasons for early mortality during the neonatal period are cardiac and respiratory complications, and central venous line infections with sepsis in GS patients [35–38]. However, high-volume centers may also report zero mortality until hospital discharge in infants with complex GS but report noticeable morbidity and recurrent surgical procedures [5].

Mortality after abdominal wall closure was the highest among patients with OCL + (25.0%) in our cohort. Three patients died of cardiac and respiratory complications long after surgical closure of the OC. The most frequent conditions limiting survival in patients with OC are associated syndromes, lung pathologies such as pulmonary dys- or hypoplasia, and cardiac malformations. With two patients who eventually died because they were treated palliatively and three patients who died after hospital discharge due to cardiac and respiratory complications, our survival rate is comparable to the data in the literature. For patients with chromosomal anomalies, the mortality is reported to be up to 40%. Generally, the two-year survival rate for OC patients is approximately 75% [18, 39, 40].

### Limitations

The limitations of our study lie in its retrospective and single-center study design. Additionally, nonsurveyed patients might have gone to other hospitals for surgical care. Interestingly, we had a high proportion of patients with complex gastroschisis (46%). When consulting the literature, the majority of publications show proportions of one complex case for every 4–9 cases of simple GS [19]. It seems that prenatally identified complex cases may pool in our institution due to the university hospital setting.

## Conclusion

In this study, we determined the incidence of secondary operations of each type of GS and OC. Complex GS and OCL + carry the highest risk to undergo secondary operations. Complex GS has a high rate for gastrointestinal related surgical procedures. The overall risk for secondary operations in simple GS and OCL − is low. The majority of procedures were performed to restore gastrointestinal function and to supply nutrition either enterally or parenterally. Gastrointestinal complications with the need for surgery substantially contribute to mortality in gastroschisis in the first year after abdominal wall closure. Mortality in patients with omphalocele, however, is not determined by gastrointestinal complications despite the high number of gastrointestinal-related surgical procedures in these patients.

These findings show that the pediatric surgical contribution remains indispensable in the treatment of patients with GS and OC after abdominal wall closure. Despite general improvements in long-term outcomes and survival, especially in patients with gastroschisis, noticeable morbidity and recurrent surgical procedures affect a high number of patients with high resource utilization. These findings will assist clinicians in managing patient care and in counseling parents with children with abdominal wall defects.

## Supplementary Information

Below is the link to the electronic supplementary material.

Supplementary file1 (DOCX 21 KB)
