# Supplementary material for: Incidence of surgical procedures for gastrointestinal complications after abdominal wall closure in patients with gastroschisis and omphalocele
Source: Pediatr Surg Int. 2021 Aug 25;37(11):1531–42. doi: 10.1007/s00383-021-04977-0 (PMC8520871; doi:10.1007/s00383-021-04977-0)
Supplement: Supplementary file 1 — Supplementary file1 (DOCX 21 KB) [file 383_2021_4977_MOESM1_ESM.docx]

**Supplementary Table 1** Initial abdominal findings at primary surgery in 35 patients with gastroschisis and 26 patients with omphalocele.

| **Intraoperative findings**  (multiple findings per patient) | **Gastroschisis** (n=35) | | **Omphalocele** (n=26) | |
| --- | --- | --- | --- | --- |
|  | Number of patients | Number of findings | Number of patients | Number of findings |
| **Mesenteric bands or Ladd’s bands**   - w atresia - w bowel stenosis - w/o constrictions | **6 (17.1%)** | **6**  2  2  2 | **1 (3.8%)** | **1**  -  -  1 |
| **Patent omphalomesenteric duct**  **Urachal fistula**  **Bowel atresia**   - Jejunum - Ileum - Ileocecal - Apple-peel atresia - Sausage-string atresia (small bowel) - Colon | **-**  **-**  **9 (25.7%)** | **-**  **-**  **12**  2  3  2  1  1  3 | **6 (23.1%)**  **2 (7.7%)**  **-** | **6**  **2**  **-** |
| **Bowel perforation**   - Birth trauma^*^ - Secondary to atresia^**^ | **2 (5.7%)** | **2**  1  1 | **-** | **-** |
| **Volvulus**^***^  **Diaphragmatic hernia**  **Other**   - Meconium impacted ileum, unused colon (ileal stenosis) - Megacolon - Long serosal defect (greater curvature of stomach, jejunum and colon) - Ileal stenosis due to adhesion to the inner lining of the omphalocele - Large gap of the ileal mesentery | **1 (2.9%)**  **-**  **3 (8.6%)** | **1**  **-**  **3**  1  1  1  -  - | **-**  **3 (11.5%)**  **1 (3.8%)** | **-**  **3**  **1**  1  1 |

w=with, w/o=without, *patient with complex birth trauma: esophageal/bowel/stomach tear, **patient with ileal atresia and perforation of jejunum, ***subtotal volvulus at abdominal wall orifice
